# Spatial evolution of international air cargo network connectivity in China: 1996–2019

**DOI:** 10.1038/s41598-025-11639-x

**Published:** 2025-07-13

**Authors:** Xu Xu, Jicheng Liu, Jialin Jiang, Bo Lin, Linna Li

**Affiliations:** 1 College of Civil Engineering and Architecture, Wenzhou University, Wenzhou, 325035 China; 2 Laboratory of Ecological Civilization and Environmental Management, Wenzhou University, Wenzhou, 325035 China; 3 Faculty of Geographical Science, Beijing Normal University, Beijing, 100875 China

**Keywords:** Air cargo transportation, Transport geography, Transport network, Connectivity, Environmental sciences, Environmental social sciences

## Abstract

The interconnection of transport infrastructure is a priority area under the Belt and Road Initiative and holds significant implications for global socio-economic development. Air network connectivity plays a vital role in shaping transport efficiency. From a global air transport network perspective, this study investigates the spatial evolution of China’s international air cargo network connectivity between 1996 and 2019. Using OAG global flight schedule data from five representative years (1996, 2002, 2008, 2014, and 2019), we apply complex network theory and graph-theoretical methods, incorporating tools such as UciNet and ArcMap. Three key indicators, weighted degree centrality, betweenness centrality, and K-core decomposition, are employed to analyze changes, evolutionary patterns, and driving factors of network connectivity. The findings reveal that with shifting global economic and policy environments, China’s air cargo network connectivity has gradually strengthened, with an increasingly hierarchical structure and more diversified and efficient network forms. Freighter and bellyhold cargo routes differ markedly in network evolution: freighter routes display a stable core-periphery structure shaped by industrial and policy factors, while bellyhold routes evolve path-dependently alongside passenger services, shifting from concentration to decentralization. This study demonstrates the dynamic restructuring of China’s international air cargo connectivity under the dual forces of globalization and regionalization. It provides theoretical insights and decision-making references for enhancing China’s air transport efficiency and supporting the implementation of the Belt and Road vision, thereby contributing to the advancement of global integration.

**Supplementary Information:**

The online version contains supplementary material available at 10.1038/s41598-025-11639-x.

## Introduction

The interconnection of transport infrastructure is both a core component and a prerequisite for the development of the Belt and Road Initiative (BRI). As a priority area of BRI cooperation, transport connectivity plays a foundational role in promoting regional economic integration by eliminating cross-border and interregional transport bottlenecks. It significantly enhances the efficiency and convenience of cross-border logistics and contributes substantially to global socio-economic development. Against this backdrop, China has entered a new stage of opening up to the world. The “Air Silk Road,” with its advantages of speed, efficiency, and convenience, is emerging as a key driver in expanding global trade and supply chain networks. It serves as a crucial pillar in supporting the implementation of the Belt and Road Initiative.

An air transport network is a type of complex network composed of airports (or cities) as nodes and air routes as edges, structured according to specific patterns. It serves as a critical framework for air transportation, representing spatial accessibility between cities and directly reflecting the level of socio-economic interactions and connectivity among them. Connectivity has emerged as a core concept in the study of air transport networks and has garnered growing academic attention in recent years. Its research spans multiple disciplines, including economic geography, air transport geography, operations research, physics, and computer science. Among these, measuring and analyzing air transport networks through the lens of hub connectivity and network integration has become a central focus in air transport geography. As the most fundamental attribute of air networks, connectivity directly influences the overall efficiency of air transportation systems^1^. It is commonly defined as the degree of convenience with which a given node is linked to other nodes within the network, serving as a key indicator of both accessibility and the capacity for socio-economic interaction^2,3^. A well-organized and reasonably distributed air network plays a vital role in advancing the development of the aviation sector.

In recent years, global air transportation has shown a gradual shift from passenger services toward cargo operations, a trend that continues to strengthen. By the end of 2024, China had 15 dedicated cargo airlines operating a total of 256 freighter aircraft. The total volume of air cargo and mail reached 8.982 million tons, of which 3.606 million tons were handled on international routes. Notably, China contributed over 30% of the global incremental growth in international air cargo during this period. This study adopts a global air transport network perspective and employs flight schedule data from 1996 to 2019. Utilizing complex network analysis and graph-theoretical methods, along with UciNet and ArcMap software, it systematically examines the evolution of China’s international air cargo network connectivity over this period, and further investigates its evolutionary characteristics and underlying driving mechanisms. The findings are of great significance for promoting the efficient development of China’s air transport sector, advancing the construction of the “Air Silk Road,” and supporting the implementation of China’s BRI.

## Literature review

Research on air network connectivity has primarily focused on the evaluation, evolution, and optimization of connectivity^4,5^. The methods commonly employed include degree centrality, closeness centrality, and shortest path analysis^5–7^. Scholars have also integrated various factors that influence connection quality, such as detour rates, transfer times, flight schedules, and aircraft types^8,9^. Based on these metrics, models such as the NetScan model^1,5^weighted connection count index, weighted feasible connection model, and weighted connection number method have been developed^5,10^. Additionally, indices like the hub connectivity index and hub international connectivity index have been proposed to evaluate connectivity at a higher level^11^. These approaches allow for a multi-dimensional assessment of air connectivity^7,12^.

Since 2019, new studies have examined the impact of the COVID-19 pandemic on air networks, particularly from the perspectives of network resilience and optimization^12–15^. The application of network analysis tools such as Python, Pajek, and Gephi, combined with GIS platforms, has further enhanced the analytical power and spatial visualization of connectivity analysis^13,16,17^.

In recent years, research in the air cargo sector has increasingly focused on enhancing airport agility, automating cargo logistics, and developing infrastructure for advanced air mobility. Digital transformation and improved information sharing have enhanced airport responsiveness and reduced inefficiencies in logistics operations^18,19^. Furthermore, the emergence of vertiports is regarded as a crucial advancement in modern air cargo, supporting the deployment of electric vertical takeoff and landing (eVTOL) aircraft^20^. These technological innovations are helping airports improve efficiency and reinforce the resilience and competitiveness of global supply chains.

A growing body of literature highlights the importance of multimodal coordination and spatial integration in strengthening overall transport connectivity. Recent research emphasizes that aligning train and flight schedules can significantly enhance intermodal efficiency, offering valuable guidance for the integration of multimodal strategies in air cargo network design^21^. It has also been shown that the spatial layout of the built environment plays a key role in shaping connectivity patterns within multimodal systems, particularly in optimizing airport access and urban logistics^22^.

Although existing studies provide strong theoretical and methodological support for analyzing air transport network connectivity^4,13,16^certain gaps remain. First, while China’s international air network has significantly expanded over the past decade, few studies have adopted a long-term perspective, limiting understanding of how key nodes evolve under the influence of policies, technological advancements, and geopolitical changes^17^. Second, most existing research emphasizes air passenger transport, neglecting the distinct functions and structural characteristics of air cargo networks. Air cargo plays a fundamental role in global supply chains and regional economies and is closely tied to economic development. The lack of sustained, long-term studies on air cargo networks hinders understanding of their spatial-temporal dynamics across different economic cycles and policy regimes, thereby limiting insights into network resilience and weakening the basis for evidence-based planning. However, a systematic analysis of its network connectivity characteristics and evolutionary mechanisms is still lacking^23^.

Given the importance of air cargo connectivity within global air transport networks, this study adopts a long-term international perspective. Using aviation schedule data from 1996 to 2019, it systematically measures and analyzes the evolution of China’s air cargo connectivity. By comparing multi-year historical data, this research examines the development patterns of key air cargo hub cities, route corridors, and international connectivity structures. The findings contribute to a better understanding of China’s global integration, reveal the spatial dynamics of its international relations, and offer references for enhancing trade facilitation. Furthermore, it provides theoretical support for informed decision-making in the air cargo industry both domestically and internationally.

## Methods and data source

### Data source

This study uses data from the Schedules Analyser, an online analytical tool provided by the Official Aviation Guide (OAG), a global leader in flight schedule data. The dataset is comprehensive, authoritative, and reliable, covering multiple temporal cross-sections required for this research. It includes all routes operated by domestic and international airlines that maintain cargo connections with China, allowing for the analysis of temporal trends and comparisons across years. Key parameters include flight number, airline, scheduled departure and arrival times, origin and destination cities, flight frequency, maximum cargo capacity, and mode of cargo transport (bellyhold or freighter). A total of 190,615 route records corresponding to the five selected years were extracted from the database. However, the OAG database only provides scheduled air service data from 1996 onwards, and the data type is limited to planned flight schedules, which reflect the theoretical cargo capacity of aircraft rather than actual freight volumes. Since actual freight data involve commercial confidentiality among airlines, it is difficult to obtain large-scale, long-term time series data, which poses certain constraints on in-depth studies of air cargo networks. Therefore, in the absence of actual data, scheduled data remain highly applicable for revealing the long-term evolutionary trends and structural characteristics of air cargo networks.

### Study period

**Fig. 1:**
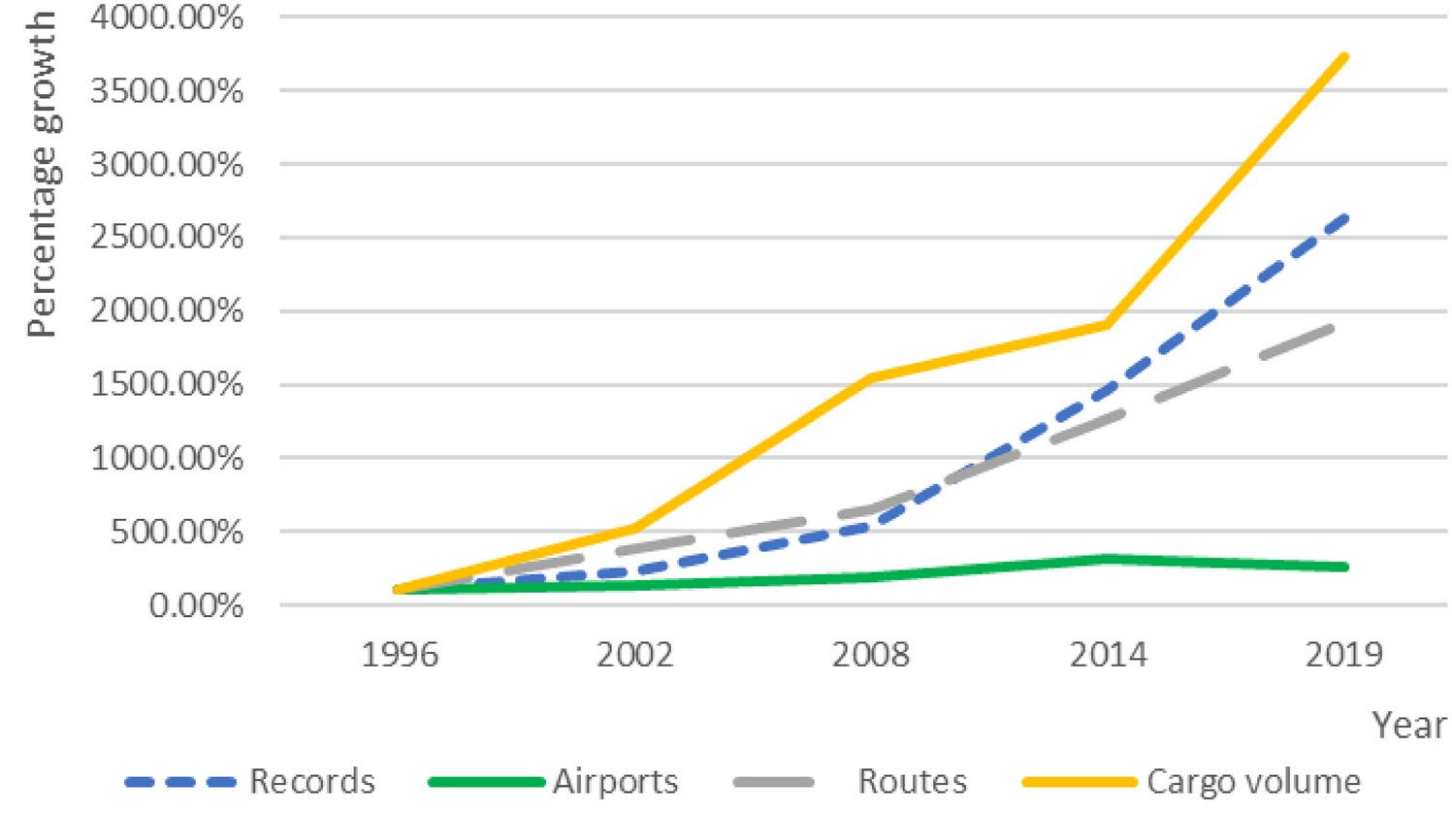
Trend of China’s International Air Cargo Development (1996–2019).

The study period covers 24 years, from 1996 to 2019. Long-term data in the field of air transportation remains limited. Most existing studies in China rely on short-term datasets, which restrict the scope of longitudinal analysis. Few studies have utilized this specific dataset, and there is a lack of research focusing on long-term air cargo network development. In the late 1990 s, China’s international air cargo sector was still in its early stages. This study selects 1996 as the starting point and uses panel data from five key years: 1996, 2002, 2008, 2014, and 2019. These years were chosen to avoid disturbances from external shocks such as the 2003 SARS outbreak, the 2009 global financial crisis, and the COVID-19 pandemic starting in 2020. This approach ensures the analysis reflects more stable patterns over time. The dataset shows a clear growth trend. China’s international air cargo sector has experienced a multiple-fold—or even several dozen-fold—increase in terms of flight frequency, number of airport nodes, route count, and cargo volume (Fig. 1). The number of cargo transport records increased from 3,880 in 1996 to 102,186 in 2019. Total cargo volume rose significantly from 620,000 metric tons to 23.13 million metric tons. The number of connected cities expanded from 123 to 322, while cities served by dedicated freighters grew from 30 to 93(Table 1). Notably, the number of network nodes saw a significant surge in 2014, followed by a slight decline in 2019.

**Table 1:** Overview of china’s international air cargo network (1996–2019).

Year	Records	Airports	Routes	Cargo volume (tons)	Freighter airports	Freighter cargo volume (tons)
1996	3,880	123	182	620,387	30	441,823
2002	8,898	163	694	3,214,327	48	1,650,578
2008	21,076	226	1188	9,615,172	88	6,413,337
2014	56,639	380	2300	11,828,727	95	7,305,369
2019	102,186	322	3484	23,133,941	93	8,236,463

*Data source: OAG Online Schedules Analyser*.

### Research methods

From the perspective of transport geography, connectivity is defined as how easily an airport is linked to other airports within the network^24^. It is considered a supply-side measure of how effectively an airport functions as a hub. The quality of connectivity directly affects the efficiency of the overall air transport network. In the context of the global aviation network, international connectivity describes the degree to which domestic nodes are linked to foreign nodes. It reflects the essential function of hub cities in linking domestic and international flows. International connectivity has two dimensions: connections from domestic nodes to foreign ones, and vice versa. Due to the symmetrical nature of air networks, these two directions are generally considered equivalent. Higher levels of international connectivity indicate closer integration of a country’s air transport system with the global network^3^.

This study applies complex network theory and graph-based analysis. UciNet, ArcMap, and relevant statistical tools are used to assess the spatial structure of air cargo connectivity. Three key indicators are used: degree centrality, betweenness centrality, and k-core decomposition. These metrics reflect the strength and breadth of a city’s links, its role in cargo transfer, and its position within the broader network hierarchy^25^. During data processing, airports in the same city are aggregated, treating the city as a single node. It should be noted that while this approach helps maintain consistency in network analysis and reduces computational complexity, it may obscure structural differences and functional specialization among airports within the same city, especially in cases where airport roles are clearly divided. This simplification may affect the interpretation of a city’s actual position in the air cargo network.

In major international hub cities, airport systems often exhibit several common patterns of functional division. The first is cargo-passenger separation, as seen in Shanghai, where Pudong Airport handles most international cargo and part of the international passenger traffic, while Hongqiao Airport focuses on domestic flights and limited short-haul international routes. A similar pattern exists in Mumbai. The second is international-domestic segmentation, such as in Tokyo, where Haneda Airport mainly serves domestic routes and Narita focuses on international services. The third is a primary-secondary hub structure, as in London, where Heathrow functions as the main international hub, while Gatwick and Luton serve low-cost and regional carriers. The fourth is spatial coverage optimization, with airports like Beijing Daxing and Chengdu Tianfu extending service coverage to alleviate congestion or reach peripheral regions.

In this study, multiple airports within the same city were aggregated at the city level. This approach was adopted to align with the research focus on the evolution of international air cargo connectivity at the urban scale, rather than on airport-level dynamics within cities.

Flight routes are classified into two types: freighter routes and belly cargo routes. Distinguishing between the two is of significant analytical value. Freighter routes operate with greater autonomy and stable capacity, better reflecting the actual flow of goods shaped by intercity industrial division and supply chain collaboration. In contrast, belly cargo transport is largely constrained by passenger network layouts and flight frequencies, making it less capable of independently capturing cargo demand patterns. Therefore, analyzing freighter and belly cargo routes separately and in combination allows for a more comprehensive understanding of the spatial structure of regional economic linkages and industrial cooperation within the air cargo network.

#### Weighted degree centrality

In an air transport network, the degree centrality of a city refers to the total number of connections it maintains with other cities, reflecting its connectivity range and intensity. The weighted degree centrality considers not only the number of connected routes but also the strength of these connections. This metric captures both the breadth and intensity of intercity links.

The formula is as follows:

1 $$w_i=\left(\sum_j^N\:a_{ij}\right)^\Game\:\times\left(\sum_j^N\:w_{ij}/\sum_j^N\:a_{ij}\right)^{1-\Game}$$

Where:

*a*_ij_ indicates whether there is a route between cities *i* and *j* (unweighted); *w*_ij_ represents the intensity of the route between cities *i* and *j* (weighted); *w*_i_ is the weighted degree centrality that incorporates both connection strength and range; *∂* is a parameter that adjusts the relative importance of intensity and range. In this study, ∂ is set to 0.5, giving equal weight to both. Connection intensity reflects the density of routes between cities, while range refers to the spatial extent of a city’s connections. Both constitute key dimensions for evaluating the functional role of a city node within the network. Connection range reflects a city’s spatial reach and connectivity scope, helping to reveal its openness and gateway position within regional or global networks. In contrast, connection strength indicates the intensity of interaction with specific cities, representing the actual volume and frequency of cargo or passenger exchanges. While the former emphasizes network coverage, the latter highlights the concentration of hub-level connections—corresponding respectively to a city’s range of connections and depth of engagement. Therefore, when assessing a city’s hub function and economic role within the regional air network, both range and intensity should be regarded as equally important and complementary indicators.

To assess the robustness of the results, a sensitivity analysis of the β parameter in weighted degree centrality was conducted. Specifically, based on all types of air cargo network data for 2008 and 2019, β was set to 0 (considering only connectivity range), 0.5 (giving equal weight to network structure and connection intensity), and 1 (considering only connectivity intensity), and node centrality rankings were compared under these three scenarios. The results show that the rankings of major hub cities remain generally stable across different β values, with only a few nodes exhibiting significant changes (see Appendix Tables 1 and 2). It should be noted that the Spearman rank correlation coefficient between β = 0 and β = 1 is relatively low (0.411 in 2008 and 0.408 in 2019; see Appendix Table 3), as these two cases represent the extreme attributes of network range and cargo flow intensity, respectively, making substantial differences in rankings reasonable. Importantly, the correlations between β = 0.5 and both extremes are relatively high, indicating that under the main analytical parameter, the network structure and the identification of key hubs remain robust, and the spatial hierarchy is not substantively affected. Overall, the sensitivity analysis further confirms the reliability of the main conclusions.

**Table 2:** K-core network evaluation parameters for different cargo transport modes.

Year	Freighter routes			Bellyhold cargo routes			All routes		
	K-value	No. of K-core nodes	No. of K-core edges	K-value	No. of K-core nodes	No. of K-core edges	K-value	No. of K-core nodes	No. of K-core edges
1996	3	30	79	2	45	120	3	123	182
2002	3	48	148	6	116	480	6	163	694
2008	5	88	364	6	226	872	7	226	1188
2014	5	95	402	8	208	1194	10	380	2300
2019	5	93	450	15	309	3292	15	322	3482

*Data source: OAG Online Schedules Analyser*.

**Table 3:** Network centralization of connectivity intensity and range.

Year/Network type	1996	2002	2008	2014	2019
Freighter network	26.08%	9.68%	13.09%	9.78%	9.52%
Bellyhold cargo network	14.62%	5.30%	4.31%	4.67%	2.88%

*Data source: OAG Online Schedules Analyser*.

#### Betweenness centrality

A city’s transfer capacity in the air cargo network is measured by betweenness centrality. Unlike connection range and intensity, this indicator focuses on the role a city plays between other cities. The more shortest paths that pass through a city, the stronger its transfer capacity.

In air transport geography, betweenness centrality measures how frequently a node lies on the shortest paths between other nodes, reflecting its potential role as a transfer hub. A city with higher betweenness centrality often serves as a key intermediary in cargo flow, linking otherwise unconnected nodes through indirect connections. This intermediary position not only contributes to improved overall network efficiency but also indicates stronger cargo handling capacity, greater logistical coordination potential, and possible influence on cargo throughput and average dwell time within the network.

2 $$BC_K=\frac{\text{2}}{{n{^2-3n+2}}}\sum_{i=1,j\neq\:k}^n\left(\sum_{j\neq\:k}^n\:\frac{\delta^{k}_{ij}}{\delta_{ij}}\right)$$

Where:

*n* is the number of cities in the network; δ_ij_ is the number of shortest paths between cities *i* and *j*; δ^k^_ij_ is the number of those paths that pass through city *k*.

#### K-core decomposition

K-core decomposition is a method used to analyze the hierarchical structure of a global network by progressively removing lower-degree nodes. For each year’s air cargo network, nodes are removed step by step starting from K = 1, then K = 2, 3, and so on, until no subnetwork remains. The largest value of K before the network disappears is defined as the maximum core. The subnet formed by the remaining airports represents the core structure of the network.

This method simplifies the full network into a backbone structure, helping to reveal its internal layers. The network is recursively filtered at K = 1, K = 2, …, with the final network consisting of the largest K-core, where K represents the degree of the participating nodes. This allows the identification of cohesive subgroups that may not be the most densely connected, but still share similar network roles or behaviors, like factions in social networks.

## Overall network characteristics

### Characteristics of complex network systems

The average clustering coefficient, average path length, power-law exponent (b), and exponential fitting parameter (b) were calculated for the years 1996, 2002, 2008, 2014, and 2019. In 1996, the power-law exponent was 1.29, which is slightly low. In 2002, 2008, 2014, and 2019, the exponent ranged from 2.3 to 2.7. This range matches the typical values for scale-free networks. For all years, the exponential fitting parameter was 1.0, indicating a poor fit to the exponential distribution. Overall, China’s international air cargo network, especially after 2002, shows clear scale-free characteristics. Hub cities have exceptionally high connectivity.

### Network connectivity

Based on the K-core decomposition method, the hierarchical structure and connectivity of the air cargo network were analyzed through recursive filtering (Table 2).

(1) The air cargo network has grown notably in both hierarchical depth and structural complexity. The K-core value of the overall network increased from 3 in 1996 to 15 in 2019. The growth was particularly notable for belly cargo routes, with K values rising from 2 to 15, while the increase for freighter routes was more modest, from 3 to 5.

(2) Network connectivity has improved significantly. The number of edges in the K-core network increased more than 19-fold over the 24-year period. For belly cargo routes, the number of K-core edges increased sharply from 86 to 3,292, whereas for freighter routes, the growth was slower, rising from 79 to 450.

(3) Significant expansion in the geographic scope of the core network. Over the past two decades, the number of nodes in the K-core network has nearly tripled, primarily driven by the expansion of belly cargo routes. The number of nodes in this type of network increased almost sevenfold, whereas the geographic coverage of the freighter network remained limited, with its node count growing from 30 to 93. This growth was particularly evident during the 2002–2008 period. From 2014 to 2019, the number of nodes for both the overall network and the belly cargo network slightly declined, indicating a marginal contraction in the network’s geographic scope.

From the perspective of overall connectivity, the period from 2002 to 2008 witnessed the most remarkable changes in terms of network expansion, improved connectivity, and hierarchical evolution. Although this period coincided with global challenges, such as the financial crisis, volatile oil prices, and rising food costs, the 2008 Beijing Olympics played a crucial role in boosting air cargo through enhanced logistics demand. Historically, the Olympics have had a positive impact on host cities’ air transport markets. In addition, the “Open Skies” agreement signed between the U.S. and Europe in 2007 had profound effects on the global aviation industry. This agreement encouraged countries, including China, to seek new trade corridors, prompting structural realignment of international air networks. These factors collectively contributed to the increase in node cities and significantly enhanced network accessibility.

Moreover, in the early 21 st century, China’s international air passenger transport experienced considerable growth. Given the strong reliance of belly cargo on passenger flights, this led to substantial improvements in both network connectivity and geographic reach. In contrast, the freighter network maintained a stable K-value of 5 from 2008 to 2019, with a modest 6% increase in K-core nodes, indicating a steady optimization of the overall network structure.

### Network centralization analysis

Between 1996 and 2019, the graph centrality of China’s international air cargo network demonstrated a significant decline, as the network shifted from a highly centralized structure to a more decentralized form (Table 3). The network structure evolved from a monocentric model to a polycentric one. However, the belly cargo and freighter route networks demonstrated different evolutionary patterns during this period. The graph centrality of the belly cargo network decreased sharply from 14.62 to 2.88%, marking a significant reduction. This suggests that the spatial organization of these routes shifted from being dominated by a few hub airports to a more balanced development involving multiple nodes.

The freighter network showed a rapid trend of decentralization around the turn of the century. This shift, observed between 1996 and 2002, may have been driven by the surge in foreign trade following China’s accession to the WTO. During this period, airlines increasingly adopted multi-point deployment strategies for launching international freighter routes, breaking the previous monopoly held by a few central hubs. In the following decade, the graph centrality of the freighter network remained relatively stable at around 10%, indicating that its spatial structure had entered a relatively mature stage.

## Evolution of cities

### Based on connectivity range and intensity

Degree centrality was used to measure both the intensity and range of each airport’s external connections. This allowed us to classify airports by their functional role. Overall, the freighter network follows a pronounced hub-and-spoke pattern, while the bellyhold cargo network exhibits a monocentric structure.

**(1)Freighter routes**.

The freighter network evolved toward a single hub–multiple nodes pattern. Shanghai serves as the dominant hub. It connects globally via secondary hubs such as Anchorage, Frankfurt, and Seoul.

**Fig. 2:**
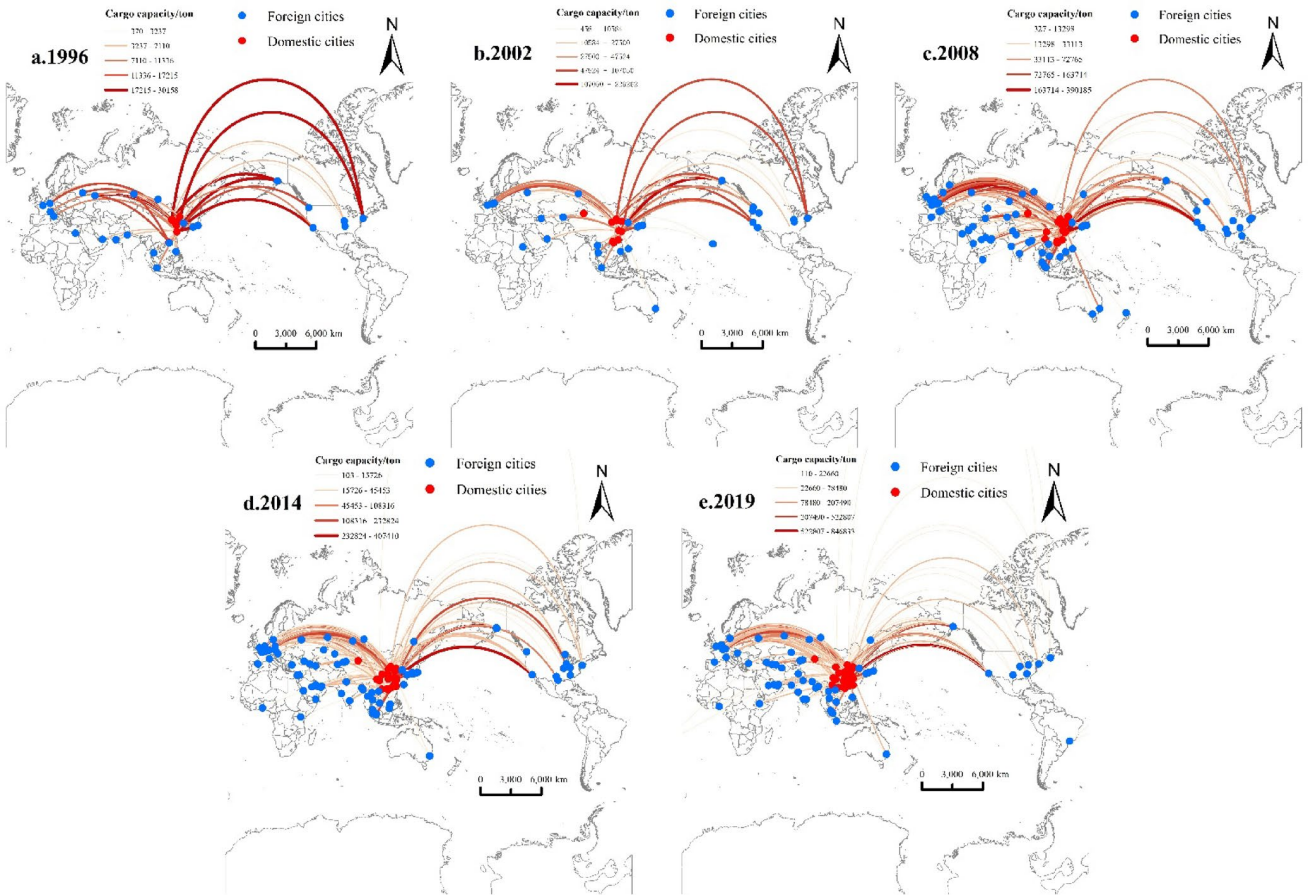
Evolution of China’s International Air Cargo Network (Freighter Routes) from 1996 to 2019.

Key features are shown in Fig. 2; Table 4:

**Table 4:** Top 20 cities by degree centrality (Freighter Routes).

Ranking	1996		2002		2008		2014		2019	
	City	Degree Centrality	City	Degree Centrality	City	Degree Centrality	City	Degree Centrality	City	Degree Centrality
1	Shanghai	28.571	Shanghai	9.911	Shanghai	13.266	Shanghai	9.882	Shanghai	9.684
2	Beijing	19.682	Beijing	3.99	Beijing	2.402	Seoul	1.352	Hong Kong	1.647
3	Novosibirsk	9.513	Anchorage	3.413	Seoul	2.035	Anchorage	1.247	Zhengzhou	1.501
4	New York	8.152	New York	1.061	Anchorage	1.545	Frankfurt	1.205	Anchorage	1.431
5	Ulyanovsk	5.666	Frankfurt	0.978	Frankfurt	1.391	Beijing	0.947	Guangzhou	1.427
6	Tianjin	5.549	Seoul	0.904	Osaka	1.269	Chicago	0.91	Chicago	1.391
7	Anchorage	5.522	Los Angeles	0.878	Amsterdam	1.236	Hong Kong	0.89	Seoul	1.255
8	Moscow	5.111	Tokyo	0.803	Hong Kong	1.197	Guangzhou	0.888	Los Angeles	1.017
9	Shenzhen	5.008	Chicago	0.799	Tianjin	1.18	Luxembourg	0.806	Frankfurt	0.993
10	Tokyo	5.005	Shenzhen	0.76	Tokyo	1.015	Amsterdam	0.799	Moscow	0.965
11	Cairo	4.087	Osaka	0.708	Shenzhen	1.01	Los Angeles	0.759	Tokyo	0.935
12	Osaka	3.709	Moscow	0.69	Los Angeles	0.944	Zhengzhou	0.735	Tianjin	0.912
13	Shenyang	2.749	Paris	0.673	Xiamen	0.94	Tianjin	0.674	Amsterdam	0.868
14	Frankfurt	2.239	Hong Kong	0.536	Chicago	0.893	Novosibirsk	0.637	Luxembourg	0.802
15	Chicago	1.924	Seattle	0.499	Luxembourg	0.857	Moscow	0.599	Taipei	0.651
16	Irkutsk	1.601	Tashkent	0.379	Paris	0.761	Tokyo	0.55	Osaka	0.608
17	Dubai	1.583	Ontario	0.364	Moscow	0.736	Osaka	0.507	Beijing	0.546
18	Milan	1.465	Luxembourg	0.354	Guangzhou	0.724	Taipei	0.501	Baku	0.464
19	Seattle	1.401	Bangkok	0.303	Nanjing	0.666	Singapore	0.476	Chongqing	0.455
20	Brussels	1.369	Singapore	0.288	Macau	0.659	Chongqing	0.339	Shenzhen	0.431

A. Rise of Chinese Cities.

Shanghai holds the core position in the global freighter network. Zhengzhou entered the top 12 in 2014, and climbed to third place by 2019. This is closely related to Cargolux’s strategic eastward shift following the launch of the BRI. In 2014, the Cargolux China project was initiated, marking its formal and deep integration into the Eurasian air freight network. Shenzhen fell from ninth in 1996 to twentieth in 2019, as cargo was diverted to nearby Hong Kong. Due to the BRI and the expansion of the cargo area at Beijing Capital Airport, Chongqing and Beijing entered the top 20 in 2019.

B. Diversification of Traditional Hubs.

Anchorage remained in the top 10, driven by trans-Pacific transfer demand. Frankfurt and Chicago, anchored by UPS and DHL operations, stayed within the top 20 despite some rank fluctuations. Japanese hubs saw continued decline.

C. Emergence of New Nodes.

Baku entered the top 20 in 2019, propelled by Eurasian transit demand via the Caspian corridor. Luxembourg, home to Cargolux, one of the largest freighter airlines, consistently ranked in the top 20.

Over the 1996–2019 period, the absolute values of centrality for core cities decreased. This change marks a transition from a “single-hub focus” to a more balanced “multi-hub system,” illustrating the diffusion phase of network maturity in transport geography.

**(2)Bellyhold cargo routes**.

The evolution of bellyhold cargo routes is largely shaped by the structure of international passenger networks, demonstrating path-dependent growth patterns shaped by passenger service reliance. Traditional hubs such as Beijing and Shanghai have maintained central positions due to their historically accumulated passenger route resources. Meanwhile, the evolution of bellyhold cargo networks shows a lagged response to changes in urban economic development, driven by this path dependence.

**Fig. 3:**
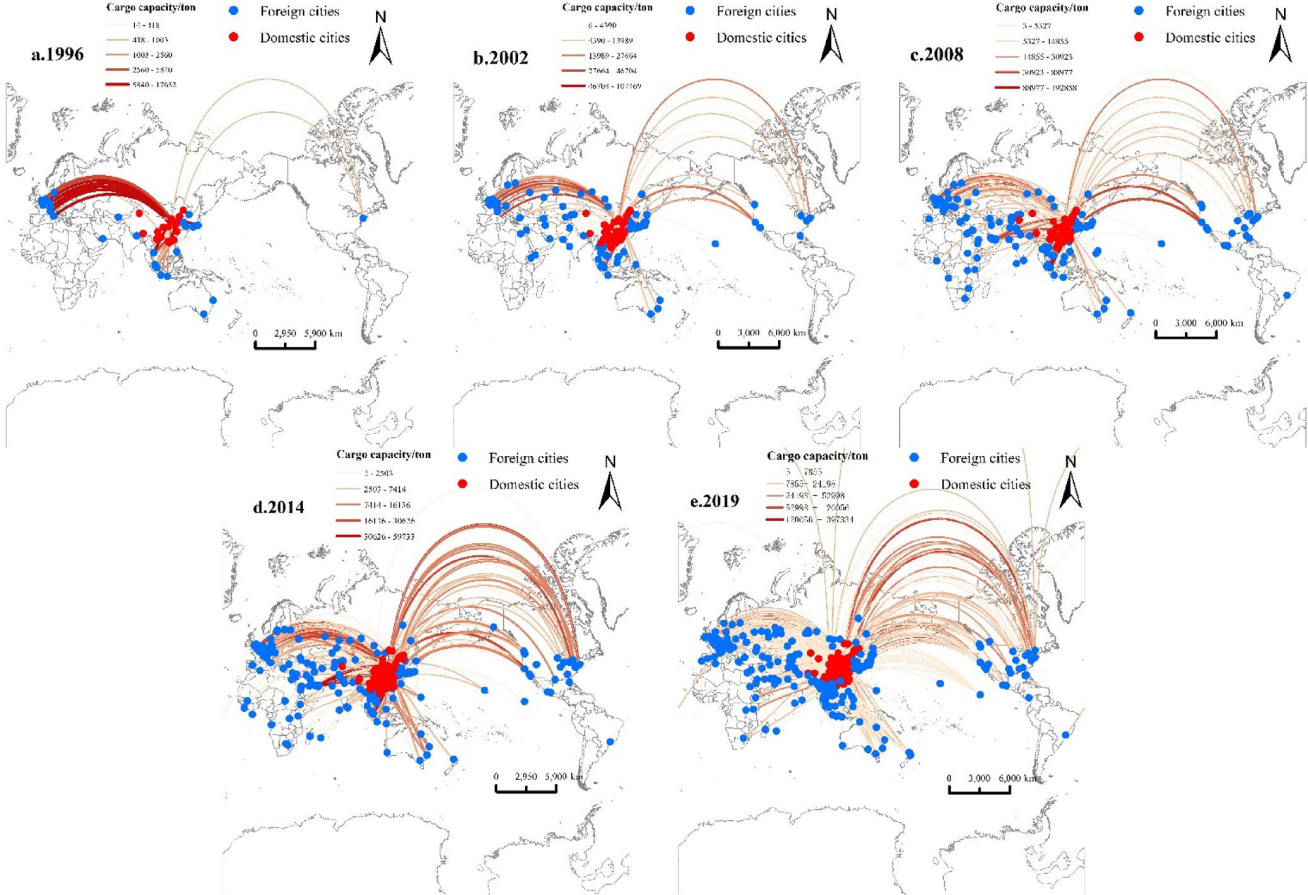
Evolution of China’s International Air Cargo Network (Bellyhold Cargo Routes) from 1996 to 2019.

Several key characteristics emerge as shown in Fig. 3; Table 5:

**Table 5:** Top 20 cities by degree centrality (Bellyhold cargo Routes).

Ranking	1996		2002		2008		2014		2019	
	City	Degree Centrality	City	Degree Centrality	City	Degree Centrality	City	Degree Centrality	City	Degree Centrality
1	Beijing	15.382	Beijing	5.429	Shanghai	4.34	Shanghai	4.777	Shanghai	3.362
2	Hong Kong	7.107	Shanghai	4.103	Beijing	1.478	Beijing	4.305	Beijing	2.309
3	Shanghai	4.796	Hong Kong	2.092	Seoul	0.969	Seoul	1.865	Guangzhou	1.658
4	Zurich	4.545	Tokyo	1.792	Hong Kong	0.592	Taipei	1.748	Bangkok	1.328
5	Guangzhou	3.365	Seoul	1.222	Tokyo	0.525	Guangzhou	1.335	Seoul	0.924
6	Frankfurt	2.97	Osaka	0.817	Singapore	0.447	Hong Kong	1.076	Tokyo	0.92
7	Bangkok	2.676	Bangkok	0.66	Frankfurt	0.425	Singapore	0.834	Singapore	0.687
8	Xiamen	2.509	Singapore	0.627	Guangzhou	0.403	Tokyo	0.753	Osaka	0.549
9	Seoul	2.449	Guangzhou	0.515	Osaka	0.388	Dubai	0.725	Taipei	0.528
10	Shenyang	2.083	Frankfurt	0.478	Anchorage	0.387	Bangkok	0.558	Shenzhen	0.437
11	Rome	1.574	San Francisco	0.459	Amsterdam	0.387	Amsterdam	0.552	Xiamen	0.426
12	Milan	1.574	Dalian	0.396	Tianjin	0.348	Shenzhen	0.469	Chengdu	0.396
13	Paris	1.545	Macau	0.36	Los Angeles	0.281	Moscow	0.447	Kuala Lumpur	0.38
14	Tokyo	1.541	Xiamen	0.36	Shenzhen	0.275	Frankfurt	0.417	Los Angeles	0.339
15	Shantou	1.299	Paris	0.319	Xiamen	0.268	Chengdu	0.388	Phuket	0.337
16	Singapore	1.024	Nagoya	0.3	Chicago	0.254	Qingdao	0.366	Kunming	0.32
17	Amsterdam	0.837	Kunming	0.298	Luxembourg	0.215	Hangzhou	0.349	Hong Kong	0.299
18	Copenhagen	0.72	Qingdao	0.275	Paris	0.204	San Francisco	0.311	Wuhan	0.293
19	London	0.689	Xi’an	0.241	Moscow	0.202	Paris	0.285	Hangzhou	0.276
20	Tianjin	0.65	Amsterdam	0.233	Macau	0.195	Kaohsiung	0.274	Dubai	0.257

First, the rise of Chinese cities is notable. Shanghai rose from third place in 1996 to consistently rank first after 2002, emphasizing its role as China’s international hub. In contrast, Beijing’s dominance in 1996 dropped sharply after 2002, likely due to a strategic shift at Capital Airport favoring passenger services, while the opening of Pudong Airport in 1999 strengthened Shanghai’s cargo capacity. Benefiting from export-oriented manufacturing in the Pearl River Delta, cross-border e-commerce, and the Western Development Policy, Guangzhou, Shenzhen, and Chengdu moved up in the rankings after 2010.

Second, hub competition in East Asia has strengthened. Seoul has consistently remained in the top five since 2002, supported by Korea’s electronics exports and Incheon Airport’s role as a regional transshipment hub. Tokyo and Osaka, however, declined in rank due to Japan’s prolonged economic stagnation and the prioritization of passenger services at Narita and Haneda Airports. Due to diversion effects from Guangzhou and Shenzhen and regional aviation policy changes, Hong Kong fell from second place in 1996 to 17th in 2019.

Third, Southeast Asia and emerging markets have gained prominence. Bangkok and Singapore have remained in the top 20, driven by regional manufacturing and transshipment demands. Dubai entered the rankings in 2014 at 13th, benefiting from Emirates Sky Cargo’s expansion and its role as a hub linking Europe, Asia, and Africa.

Finally, traditional European hubs have declined. Fragmentation in Europe’s air cargo market and rising competition from Middle Eastern and Asian hubs resulted in cities like Zurich and Frankfurt to drop out of the top 20 by 2019. Meanwhile, Amsterdam and Paris experienced continuous decline, influenced by EU air market consolidation and the growing dominance of low-cost carriers that crowded out all-cargo operations.

### Based on transshipment function

The transshipment capacity of air transport hub cities is primarily measured by betweenness centrality. This indicator evaluates the extent to which a city lies on the shortest paths between other cities. A higher value indicates that more shortest paths pass through the node, suggesting stronger transshipment capacity and improved cargo routing efficiency through the hub.

**(1)Freighter routes**.

An analysis of the betweenness centrality of freighter routes reveals significant changes in the absolute values of betweenness centrality for different cities between 1996 and 2019, with an overall downward trend, as shown in Table 6. This indicates that the network has shifted from a highly centralized structure to a more dispersed one.

**Table 6:** Top 20 cities by betweenness centrality (Freighter Routes).

Ranking	1996		2002		2008		2014		2019	
	City	Betweenness	City	Betweenness	City	Betweenness	City	Betweenness	City	Betweenness
1	Shanghai	64.717	Shanghai	78.465	Shanghai	69.577	Shanghai	58.398	Shanghai	43.357
2	Beijing	29.027	Beijing	16.163	Shenzhen	10.685	Guangzhou	21.841	Zhengzhou	17.378
3	New York	17.482	Seoul	13.338	Seoul	10.596	Hong Kong	13.325	Guangzhou	16.448
4	Novosibirsk	16.225	Moscow	4.334	Beijing	9.357	Seoul	8.812	Seoul	9.927
5	Shenzhen	7.188	Tokyo	4.255	Tianjin	8.004	Zhengzhou	8.683	Hong Kong	8.304
6	Ulyanovsk	4.557	Baku	4.255	Moscow	7.249	Beijing	5.26	Kunming	7.549
7	Tianjin	3.637	Osaka	1.608	Guangzhou	5.991	Taipei	5.105	Shenzhen	5.96
8	Shenyang	3.637	Hong Kong	1.608	Osaka	3.806	Tianjin	2.869	Tianjin	5.771
9	Chicago	3.448	Nagoya	1.019	Xiamen	2.626	Chongqing	2.576	Taipei	5.545
10	Dalian	3.325	Chicago	0.849	Tokyo	2.534	Dhaka	2.427	Wuhan	4.983
11	Irkutsk	2.709	Subic Bay	0.849	Chengdu	2.348	Hangzhou	2.105	Anchorage	3.817
12	Hong Kong	1.642	Anchorage	0.849	Dhaka	2.299	Chengdu	1.805	Chongqing	3.54
13	Brussels	1.359	Shenzhen	0.801	Frankfurt	2.207	Tokyo	1.706	Ho Chi Minh City	3.51
14	Subic Bay	1.359	Memphis	0.54	Anchorage	2.136	Frankfurt	1.66	Tokyo	3.173
15	Anchorage	1.359	Xiamen	0.131	Amsterdam	1.696	Almaty	1.405	Singapore	2.983
16	Cairo	0.774	Portland	0.079	Nanjing	1.617	Amsterdam	1.325	Osaka	2.869
17	Milan	0.774	New York	0.079	Chicago	1.518	Krasnoyarsk	1.233	Baku	2.665
18	Dubai	0.774	Ontario	0.079	Delhi	1.498	Los Angeles	1.215	Chicago	2.634
19	Osaka	0.585	Nadi	0.079	Hong Kong	1.352	Luxembourg	1.152	Urumqi	2.197
20	Tokyo	0.585	San Francisco	0.079	Bangkok	1.109	Osaka	0.978	Bishkek	2.095

Domestically, Shanghai has consistently held its position as a global air freight hub, although its centrality has gradually weakened over the years. Beijing’s position has sharply declined since 2008, while cities like Guangzhou, Zhengzhou, Taipei, and Chengdu have shown notable growth in recent years. The rise of inland cities such as Zhengzhou, Chongqing, Urumqi, and Kunming as emerging secondary hubs reflects a significant restructuring of the air cargo network in Eurasia. This transformation is primarily driven by the BRI and the accompanying infrastructure investments, which have shifted the focus of international trade toward the interior. Among these cities, Zhengzhou stands out due to its rapid increase in betweenness centrality, signaling its growing role as a key transit hub. A critical factor behind this development is the strategic eastward shift of major cargo carriers, particularly Cargolux, which strengthened its connectivity with central China and prioritized the growth of the “Zhengzhou–Luxembourg” corridor. These changes have collectively broken the traditional coastal monopoly and fostered a new core–periphery structure centered on inland nodes.

In East Asia, Seoul has remained at the forefront, while Tokyo has continued to decline. Following the collapse of the Soviet Union and Russia’s economic transformation, combined with the land route alternatives offered by the Trans-Siberian Railway, cities like Novosibirsk (4th in 1996) and Ulyanovsk (6th in 1996) suddenly dropped out of the top 20 after 2002. Furthermore, after 2019, new Central Asian hubs have emerged due to geopolitical and economic shifts in transportation network structures, with cities like Baku (17th in 2019) and Bishkek (20th in 2019) increasingly important. While the significance and roles of different cities in the global air freight network have evolved with changing geopolitical contexts, cities like Shenzhen, Hong Kong, Seoul, and Anchorage have remained important hubs throughout these 24 years.

**(2)Bellyhold cargo routes**.

Bellyhold cargo routes, due to their reliance on passenger routes, exhibit distinct evolutionary characteristics from freighter routes, evolved through distinct phases of unipolar dominance, multicentric diffusion, and regional restructuring. Several key conclusions are presented in Table 7. Beijing maintained its leading position with an overwhelming advantage for an extended period, though its relative weight steadily declined over time. Meanwhile, East Asian hubs such as Shanghai, Hong Kong, and Seoul emerged as secondary cores. Southeast Asian nodes like Bangkok and Taipei, along with inland cities like Urumqi and Kunming, gradually gained prominence. Notably, Seoul rose to third place in 2008; Hong Kong dropped out of the top five in 2014 due to the relocation of manufacturing industries from the Pearl River Delta. Benefiting from the cross-strait direct flight policy, Taipei climbed from being unranked in 2008 to fifth place in 2019. Kunming and Urumqi have seen continuous growth in importance since 2002, becoming the most significant hubs in western China. Following the ASEAN Single Aviation Market agreement in 2015, which removed air route restrictions among member states, Bangkok, Kuala Lumpur, and Singapore all saw improvements in their transshipment capabilities after 2019. Anchorage once played a vital role in the network at the end of the 20th century, but with the development of freighter routes, reliance on bellyhold cargo routes for transshipment via Anchorage has diminished.

**Table 7:** Top 20 cities by betweenness centrality (Bellyhold cargo Routes).

Ranking	1996		2002		2008		2014		2019	
	City	Betweenness	City	Betweenness	City	Betweenness	City	Betweenness	City	Betweenness
1	Beijing	58.495	Beijing	55.791	Beijing	27.221	Beijing	46.471	Beijing	34.375
2	Shanghai	57.849	Shanghai	24.822	Shanghai	26.99	Shanghai	19.897	Shanghai	23.012
3	Novosibirsk	41.47	Hong Kong	18.477	Seoul	8.567	Seoul	12.408	Guangzhou	14.367
4	Shenzhen	7.484	Seoul	11.844	Guangzhou	6.315	Taipei	12.255	Bangkok	10.542
5	Tianjin	4.151	Urumqi	5.248	Hong Kong	5.467	Guangzhou	10.061	Taipei	6.842
6	Shenyang	4.151	Bangkok	4.959	Urumqi	5.23	Urumqi	10.025	Kunming	5.103
7	Subic Bay	3.844	Macau	3.895	Shenzhen	3.078	Chengdu	6.05	Shenzhen	4.906
8	Brussels	3.844	Kunming	3.409	Kunming	2.432	Hong Kong	4.943	Chengdu	4.426
9	Anchorage	3.844	Dalian	2.594	Dubai	1.718	Kaohsiung	3.965	Singapore	4.111
10	Chicago	3.844	Osaka	2.436	Moscow	1.478	Singapore	3.897	Urumqi	3.974
11	Tokyo	3.844	Moscow	2.346	Singapore	1.397	Ulaanbaatar	2.376	Seoul	3.897
12	Memphis	3.844	Harbin	2.095	Macau	1.259	Paris	2.171	Hong Kong	3.319
13	New York	3.844	Tianjin	2.063	Tokyo	1.052	Jinan	2.015	Sanya	3.28
14	Osaka	3.844	Guangzhou	1.974	Osaka	0.892	Bangkok	1.989	Osaka	2.871
15	Ulyanovsk	1.9	Hanoi	1.971	Islamabad	0.863	Taichung	1.844	Kuala Lumpur	2.231
16	Qingdao	1.032	Tokyo	1.907	Kathmandu	0.781	Macau	1.824	Chiang Mai	1.849
17	Dalian	1.032	Xi’an	1.901	Tianjin	0.773	Tianjin	1.497	Hangzhou	1.844
18	Irkutsk	0.072	Hangzhou	1.83	Xiamen	0.732	Chongqing	1.458	Xi’an	1.79
19	Delhi	0	Fuzhou	1.808	Frankfurt	0.729	Kunming	1.409	Phuket	1.627
20	Paris	0	Kathmandu	1.709	Kuala Lumpur	0.698	Islamabad	1.298	Changsha	1.352

## The evolution of air route channels

### Freighter routes

As is shown in Fig. 2, the evolution of freighter routes during this period demonstrates two key features:

(1) Spatial restructuring of the network.

i. Deepening globalization: diversified intercontinental expansion with westward shifts.

In 1996, China’s air cargo industry remained in its early development phase, largely dependent on coastal cities and overseas demand. Major routes were concentrated in North America (e.g., Shanghai → Chicago, Shanghai → Seattle), with relatively modest volumes (e.g., 4,926 tons). By 2019, key routes had shifted toward Asia, such as Shanghai → Tokyo (35,280 tons), Shanghai → Seoul (31,400 tons), and Ningbo → Osaka (36,515 tons), accompanied by a significant rise in freight volume. New routes emerged to inland U.S. hubs such as Dallas and to gateways like Los Angeles, while cargo volumes on routes like Frankfurt → Shanghai increased more than fivefold, indicating strengthened connectivity with global secondary centers.

ii. Accelerated regionalization: increased network density within Asia.

The share of intra-Asian routes, including Northeast Asia (e.g., Osaka, Seoul) and Southeast Asia (e.g., Manila, Singapore), rose from 18% in 1996 to 42% in 2019. The Ningbo → Osaka route ranked first in 2019, with 36,515 tons.

(2) Nonlinear growth in freight volume.

Cargo volumes on top freighter routes increased from 4,926 tons in 1996 (Shanghai → Chicago) to 36,515 tons in 2019 (Ningbo → Osaka), with an average annual growth rate of 9.7%, surpassing the 7.2% national air freight growth rate. This highlights increasing concentration along leading route.

### Bellyhold cargo routes

As is shown in Fig. 3, the evolution of bellyhold cargo route networks during this stage exhibits the following characteristics:

(1) Expansion of the Southeast Asian network driven by regional economic integration.

In 1996, major routes were concentrated between Hong Kong and mainland Chinese cities, such as the Hong Kong → Shenyang route (4,120 tons), with relatively low cargo volumes. At the time, Hong Kong served as China’s primary gateway for international trade and handled the majority of bellyhold cargo. Between 2008 and 2014, primary routes shifted to Shanghai → Singapore (11,928 tons) and Beijing → Dubai (12,014 tons). By 2019, the network extended toward Southeast Asia, with increased shares of passenger routes such as Phuket → Chengdu (23,700 tons) and Taipei → Shanghai (12,948 tons).

(2) Steady growth in cargo volume.

Compared to freighter routes, bellyhold cargo volumes exhibited more moderate growth, primarily supporting domestic and short-haul regional freight. The top route volume increased from 4,120 tons (Hong Kong → Shenyang) in 1996 to 23,700 tons (Phuket → Chengdu) in 2019.

### Summary

Through statistical analysis of primary air cargo routes, it is evident that China’s air cargo network has undergone a transformation over the past two decades, progressing from single-center polarization to multi-center diffusion, and eventually evolving into hierarchical coordination and regional reorganization (Table 8). This process is closely linked to China’s rapid economic growth, expansion of foreign trade, and national policy support. In addition, strategic shifts of major cargo airlines also exert a certain influence on the network. While freighter routes and bellyhold cargo routes share similarities in their development stages and primary driving factors, they differ significantly in terms of core hub cities, route distribution, cargo volume changes, and evolution mechanisms. Freighter routes primarily serve international long-haul freight, with relatively stable core hub cities and rapid cargo volume growth. In contrast, bellyhold cargo routes mainly cater to domestic and regional freight, with more significant changes in core hub cities, moderate cargo volume growth, and more evident regionalization characteristics. This “globalization → regionalization → specialization” progressive model provides a universal analytical framework for examining the dynamic evolution of air cargo networks.

**Table 8:** Analysis of the evolutionary stages of china’s international air cargo network (1996–2019).

Stage	Period	Network structure	Key driving factors	Description
First	1996–2002	Single-center polarization	Emergence of export-oriented economy	Resources highly concentrated in coastal hubs, forming a single-center network structure
Second	2002–2014	Multi-center diffusion	Impact of WTO accession	Inland nodes rise as new cargo hubs under the policy-driven expansion
Third	2014–2019	Hierarchical collaboration	Implementation of the BRI	Hierarchical and regional reorganization of the network, with specialized division of node functions

Data availability.

The author confirms that all data generated or analysed during this study are included in this published article.

Furthermore, a preliminary analysis was conducted to examine the potential competitive dynamics and interactions between freighter services and bellyhold cargo services on specific routes. Based on the original dataset, the top 20 cargo routes by freight volume were extracted for each year, separately for bellyhold and freighter services (see Appendix Tables 4 and 5). Three overlapping routes served by both modes were identified: Beijing–Paris (2002), Frankfurt–Beijing (2002), and Shanghai–Tokyo (2019). The evolution of their capacities shows the following.

(1)Beijing–Paris.

In 1996, only bellyhold service existed (3,130 tons). Freighter service was introduced in 2002 and reached 11,518 tons. Bellyhold capacity also increased to 4,620 tons. This indicates a complementary relationship between the two modes in response to growing demand.

(2)Frankfurt–Beijing.

In 2002, both bellyhold (4,908 tons) and freighter (8,393 tons) services operated in parallel. This suggests a dual-capacity configuration for meeting cargo demand.

(3)Shanghai–Tokyo.

In 2002, only bellyhold capacity was observed (4,224 tons). By 2019, bellyhold increased to 8,200 tons, and freighter capacity reached 29,135 tons. This reflects simultaneous growth under rising demand.

These observations lead to several conclusions.

First, most routes show differentiated deployment between bellyhold and freighter services. The two modes often serve different purposes in terms of route selection, market positioning, and operational objectives. This indicates a degree of functional complementarity.

Second, on major high-demand routes, bellyhold and freighter capacities tend to grow together. The expansion of freighter services does not replace bellyhold capacity. Instead, both increase simultaneously, suggesting that they meet different freight types or time requirements.

Finally, some routes may follow a pattern of “belly-first, freighter-following”. Bellyhold services are used to test demand. Once demand is strong enough, freighters are added. This hypothesis needs further verification with more time-series data and detailed market analysis.

## Conclusion

The evolution of China’s international air cargo network is essentially a geographic projection of the interaction between the restructuring of the global production system, deepening institutional openness, and upgrading of technological infrastructure. The freighter route network, initially a singular export channel, has gradually developed into a strategic logistics network supporting the global-regional dual circulation, reflecting China’s growing position in the global value chain. From the perspective of transportation geography, the evolution of China’s international air cargo network connectivity reveals the following significant scientific conclusions:

(1) Network hierarchy and connectivity enhancement:

Due to global economic integration and China’s rapid foreign trade development, the hierarchy of China’s international air cargo network has significantly increased, and connectivity has been notably enhanced. This improvement has strengthened China’s position in the global air cargo network, facilitating more efficient connections to global markets and promoting stable international trade and supply chain development. This conclusion supports the vital role of air cargo networks in regional economic integration and validates the diffusion pattern observed in the transportation geography theory of network maturity. As networks evolve, their structure shifts from centralized to decentralized, with more nodes involved, forming a more complex and efficient network system.

(2) Heterogeneous evolution of freighter and bellyhold cargo routes:

Freighter and bellyhold cargo routes exhibit significant differences in their network evolution. Freighter routes show a"core-periphery"topological pattern, with a relatively stable network structure, although the dominance of core cities gradually weakens, and more cities become part of the network, leading to a multipolar development pattern. This suggests that freighter route development is more influenced by industrial layout and policy direction, with a strong industry-driven nature. In contrast, bellyhold cargo routes exhibit “path dependency” driven by passenger services, with their network evolution strongly impacted by passenger routes, showing a trend from concentration to decentralization. This finding reveals the differing mechanisms behind the evolution of various cargo route types, offering key insights for optimizing air cargo network layouts. In practice, air cargo network planning should consider the heterogeneity between freighter and bellyhold routes and adopt differentiated strategies to improve overall network efficiency and competitiveness.

(3) Role evolution of node cities:

The role of node cities in China’s air cargo network has undergone significant changes. Cities such as Zhengzhou and Chongqing have risen to prominence as key air cargo hubs under the BRI. Meanwhile, traditional international hubs have seen their positions decline due to industrial relocation, market competition, and other factors, while emerging nodes have grown due to regional transit demands. This phenomenon indicates that the evolution of the air cargo network is closely tied to changes in the global economic landscape, regional economic policies, and industrial layout. The role evolution of node cities not only affects the structure and function of the air cargo network but also has a significant impact on regional economic development.

In conclusion, this study reveals the spatial pattern evolution principles of China’s international air cargo network connectivity and its influencing factors from the perspective of transportation geography. It provides theoretical support and decision-making references for the efficient development of China’s air transport. The data used in this research encompasses all flight plans of Chinese airlines and foreign carriers operating Chinese routes, providing a comprehensive and reliable analysis. Future studies could further explore the resilience and restructuring of air transport networks during the COVID-19 pandemic under extreme shocks, as well as the collaborative evolution mechanisms between air cargo networks and other modes of transportation.

Looking ahead, the spatial structure and connectivity of international air cargo networks will continue to be reshaped by multiple transformative forces. The integration of artificial intelligence into logistics operations is expected to significantly enhance network efficiency and responsiveness. Meanwhile, global decarbonization efforts may drive adjustments in air cargo route structures, such as adopting new propulsion technologies and reconfiguring long-haul routes to optimize carbon emissions. Additionally, the deeper integration of air cargo into multimodal transport systems will play a crucial role in improving end-to-end connectivity, especially for inland and emerging hub cities. These trends indicate that future air cargo networks will become more intelligent, sustainable, and spatially adaptive, requiring ongoing advancement in technological innovation and multimodal coordination.

It is also important to note that this study measures connectivity primarily from a supply-side perspective, using structural indicators to represent air cargo connectivity between urban nodes. This approach is widely adopted in air transport network research and helps reveal structural properties and hub positions. However, such indicators do not account for demand-side factors, including shipper-perceived service quality, cost, or time utility, all of which significantly influence routing choices and network evolution. Future studies could enrich the understanding of connectivity by incorporating demand-side dimensions through survey data, cost modeling, or behavioral simulations, thereby offering a more comprehensive view of network performance and dynamics.

In this context, future research could adopt a more detailed, airport-level perspective. It is important to examine how dominant airports within multi-airport cities affect the structural role and connectivity performance of their urban nodes. Such analysis may reveal how functional differentiation among airports within a city contributes to the spatial organization and evolution of international air cargo networks.

While this study relates the observed evolution of the air cargo network to a set of contextual factors, such as global economic shifts, national policy initiatives and major events, these explanations are primarily based on temporal correlations. The analysis does not establish formal causal mechanisms. As such, the nature of these linkages should be interpreted as background associations rather than definitive causal inferences.

Future research is encouraged to apply more rigorous causal identification methods, such as Difference-in-Differences, event study approaches, or panel data models, to empirically evaluate the specific impact of these external factors on the structure and dynamics of international air cargo networks. Incorporating such approaches would help strengthen the explanatory power and policy relevance of network evolution studies.

## Electronic supplementary material

Below is the link to the electronic supplementary material.

Supplementary Material 1

## Data Availability

The author confirms that all data generated or analysed during this study are included in this published article.
